# Expression of CD3-**ζ** on T-cells in primary cervical carcinoma and in metastasis-positive and -negative pelvic lymph nodes

**DOI:** 10.1038/sj.bjc.6690179

**Published:** 1999-03

**Authors:** T D de Gruijl, H J Bontkes, F Peccatori, M P W Gallee, Th J M Helmerhorst, R H M Verheijen, J Aarbiou, W M C Mulder, J M M Walboomers, C J L M Meijer, N van de Vange, R J Scheper

**Affiliations:** European Cancer Centre/Amsterdam Gynaecologic Oncology Group: Department of Pathology, Free University Hospital, Postbus 7057, Amsterdam, 1007 MB, The Netherlands; European Cancer Centre/Amsterdam Gynaecologic Oncology Group: Department of Obstetrics and Gynaecology, Free University Hospital, Postbus 7057, Amsterdam, 1007 MB, The Netherlands; European Cancer Centre/Amsterdam Gynaecologic Oncology Group: Department of Pathology, Antoni van Leeuwenhoek Hospital, Plesmanlaan 121, Amsterdam, 1066 CX, The Netherlands; European Cancer Centre/Amsterdam Gynaecologic Oncology Group: Department of Obstetrics and Gynaecology, Academic Medical Center Amsterdam, Meibergdreef 9, Amsterdam, 1105 AZ, The Netherlands; European Cancer Centre/Amsterdam Gynaecologic Oncology Group: Department of Medical Oncology, European Institute of Oncology, Via Ripamonti 435, Milan, 20141, Italy

**Keywords:** cervical carcinoma, cervical dysplasia, T-lymphocytes, ζ, chain

## Abstract

Lymphocytic infiltrate is often present in cervical cancer lesions, possibly reflecting an ongoing, but ineffective, immune response to the tumour. Recently, evidence has accumulated for systemically impaired T-cell functions in cancer patients, associated with decreased expression of signal-transducing zeta (ζ) chain dimer molecules on circulating T-cells and NK-cells. Here, we report on the intralesional down-regulation of ζ chain expression on T-cells in cervical carcinoma. Paraffin-embedded or snap-frozen sections from 24 different cervical cancer specimens were studied. Paraffin-embedded tumour-positive (*n* = 7) and tumour-negative (*n* = 15) pelvic lymph nodes were also included in the study. Immunostaining was performed on consecutive sections with antibodies specific for CD3-ɛ or the CD3-associated ζ chain dimer. Antigen retrieval by sodium citrate/microwave treatment was essential for ζ staining of paraffin sections. The amount of ζ positive cells was quantitated and related to the number of CD3-ɛ+ cells in corresponding tumour areas. Of the 24 cervical cancer specimens studied, ζ chain dimer expression was reduced in seven cases and strongly reduced in the other 17 samples. In tonsil control sections, CD3-ɛ and CD3-ζ were always co-expressed in almost equal numbers. Also, both tumour-negative and -positive lymph nodes showed ζ chain expression which equalled that of CD3-ɛ expression. These data indicate that a decreased expression of signal-transducing ζ molecules on tumour-infiltrating T-cells is frequent in cervical cancer. The apparently unimpaired ζ chain expression within draining lymph nodes suggests that local tumour-derived factors at the primary site are instrumental in ζ chain down-regulation. © 1999 Cancer Research Campaign


British Journal of Cancer (1999) 79(7/8), 1127–1132
© 1999 Cancer Research Campaign
Article no. bjoc.1998.0179

### Expression of CD3-ζ on T-cells in primary cervical carcinoma and in metastasis-positive and -negative pelvic lymph nodes

**TD de Gruijl[1], HJ Bontkes[1], F Peccatori[1,5], MPW Gallee[3], ThJM Helmerhorst[2], RHM Verheijen[2], J Aarbiou[1], WMC Mulder[1], JMM Walboomers[1], CJLM Meijer[1], N van de Vange[4] and RJ Scheper[1]**

European Cancer Centre/Amsterdam Gynaecologic Oncology Group: [1]Department of Pathology and [2]Department of Obstetrics and Gynaecology, Free University Hospital, Postbus 7057, 1007 MB Amsterdam, The Netherlands; [3]Department of Pathology, Antoni van Leeuwenhoek Hospital, Plesmanlaan 121, 1066 CX Amsterdam, The Netherlands; [4]Department of Obstetrics and Gynaecology, Academic Medical Center Amsterdam, Meibergdreef 9, 1105 AZ Amsterdam, The Netherlands; [5]Department of Medical Oncology, European Institute of Oncology, Via Ripamonti 435, 20141 Milan, Italy

**Summary** Lymphocytic infiltrate is often present in cervical cancer lesions, possibly reflecting an ongoing, but ineffective, immune response to the tumour. Recently, evidence has accumulated for systemically impaired T-cell functions in cancer patients, associated with decreased expression of signal-transducing zeta (ζ) chain dimer molecules on circulating T-cells and NK-cells. Here, we report on the intralesional down-regulation of ζ chain expression on T-cells in cervical carcinoma. Paraffin-embedded or snap-frozen sections from 24 different cervical cancer specimens were studied. Paraffin-embedded tumour-positive (*n* = 7) and tumour-negative (*n* = 15) pelvic lymph nodes were also included in the study. Immunostaining was performed on consecutive sections with antibodies specific for CD3-ε or the CD3-associated ζ chain dimer. Antigen retrieval by sodium citrate/microwave treatment was essential for ζ staining of paraffin sections. The amount of ζ positive cells was quantitated and related to the number of CD3-ε+ cells in corresponding tumour areas. Of the 24 cervical cancer specimens studied, ζ chain dimer expression was reduced in seven cases and strongly reduced in the other 17 samples. In tonsil control sections, CD3-ε and CD3-ζ were always co-expressed in almost equal numbers. Also, both tumour-negative and -positive lymph nodes showed ζ chain expression which equalled that of CD3-ε expression. These data indicate that a decreased expression of signal-transducing ζ molecules on tumour-infiltrating T-cells is frequent in cervical cancer. The apparently unimpaired ζ chain expression within draining lymph nodes suggests that local tumour-derived factors at the primary site are instrumental in ζ chain down-regulation.

**Keywords:** cervical carcinoma; cervical dysplasia; T-lymphocytes; ζ chain

T-cells infiltrating neoplastic lesions are believed to mediate tumour-specific immune responses (Rosenberg, 1996). However, in vitro studies of freshly isolated tumour-infiltrating lymphocytes (TIL) have shown that such T-cells often have impaired effector functions (Whiteside et al, 1992). Immunosuppressive conditions within the tumour environment, possibly dictated by cytokines such as interleukin (IL)-10 and transforming growth facter beta (TGF-β), may account for the low activation state of tumour-infiltrating T-cells (Kiessling et al, 1996). Alternatively, alterations to the signal transduction machinery within the T-cells may hamper effective activation upon recognition of (tumour-associated) antigens (Kiessling et al, 1996; Levey and Srivastava, 1996).

The loss of signal transducing zeta (ζ) chains from the T-cell receptor (TCR) complex of peripheral T-cells was first reported in tumour-bearing mice (Mizoguchi et al, 1992). ζ Chains are usually present in T-cells as disulphide-linked, transmembrane homodimers that are associated with the CD3 complex (Robey and Allison, 1995). Previous studies revealed a reduced expression of CD3-ζ chain on peripherally circulating and/or tumour-infiltrating lymphocytes from patients with colorectal cancer (Nakagomi et al, 1993; Matsuda et al, 1995; Mulder et al, 1997), renal cell carcinoma (Finke et al, 1993; Kono et al, 1996*b*), ovarian cancer (Lai et al, 1996), and melanoma (Zea et al, 1995). Moreover, inverse correlations between the level of CD3-ζ chain expression, the stage of colorectal carcinoma (Matsuda et al, 1995; Mulder et al, 1997), and the survival rate among melanoma patients (Zea et al, 1995), strongly suggest that such a reduction in CD3 ζ chain expression indeed leads to an impaired tumour-specific immunity. In line with this, the absence of ζ chains has been correlated to a reduced proliferative and cytokine production capacity of mature T-cells (Mizoguchi et al, 1992; Finke et al, 1993; Zea et al, 1995; Tartour et al, 1995; Lai et al, 1996).

In patients with colorectal carcinoma the level of CD3-ζ expression on mucosal T-cells was found to be inversely related to their distance from the tumour site (Matsuda et al, 1995). In addition, both in renal cell and in colorectal cancer patients, a higher expression of CD3-ζ was found on peripheral T-cells than on tumour-infiltrating T-cells (Finke et al, 1993; Nakagomi et al, 1993; Matsuda et al, 1995). These results indicate that the observed loss of ζ chain expression may be caused by tumour-derived factors. We have previously studied local and systemic immune parameters in patients with malignant and premalignant lesions of the cervix uteri (Cromme et al, 1993, 1995; de Gruijl et al, 1996*a*, 1996*b*). The development of cervical cancer is strongly associated with the presence of oncogenic human papillomavirus (HPV)

*Received 25 March 1998*
*Revised 30 July 1998*
*Accepted 3 August 1998*

*Correspondence to:* RJ Scheper, Department of Pathology, Free University Hospital, PO Box 7057, 1007 MB Amsterdam, The Netherlands types (IARC, 1995). The failure of the host immune system to raise effective immune responses to HPV-derived antigens is believed to play an important role in cervical carcinogenesis. This is exemplified by increased prevalences of HPV-induced cervical lesions in patients with impaired cellular immune functions such as renal transplant recipients and AIDS patients (Frazer, 1996). Recently, a reduced expression of CD3-ζ and CD16-ζ on peripheral blood lymphocytes (PBL) from women with premalignant and malignant cervical lesions was reported (Kono et al, 1996*a*). This altered ζ chain expression was related to a reduced production of tumour necrosis factor (TNF) upon polyclonal activation of peripheral T-cells.

As ζ chain loss might represent a critical factor contributing to an impaired HPV-specific T-cell-mediated immune response, we decided to investigate the in situ ζ chain expression in primary and metastatic cervical carcinoma-infiltrating lymphocytes using immunohistochemical methods.

### MATERIALS AND METHODS

### Patients and specimens

Archives of the Pathology Departments of the Netherlands Cancer Institute, the Free University Hospital and the Academic Medical Center, Amsterdam, The Netherlands, were searched for primary squamous cell cervical carcinoma specimens stored after 1994. Whenever possible, frozen and fixed material from primary tumours and the corresponding tumour-positive or -negative pelvic lymph nodes were retrieved. Twenty-five different cases were identified. Paraffin-embedded tissue samples ($n$ = 16) and frozen biopsies ($n$ = 13) from in total 24 primary tumours were collected. For five primary tumours both frozen and paraffin material was available. Paraffin blocks of tumour-negative ($n$ = 15) and of tumour-positive lymph nodes ($n$ = 7) were retrieved. After histologic and clinical file review, all cases were classified as moderately (6/25) or poorly differentiated (19/25) squamous cell cervical carcinoma and staged Ib–IIa according to the International Federation of Gynaecology and Obstetrics (FIGO) staging system. Median patient age was 47.8 years (25–78). All patients had undergone radical hysterectomy with pelvic lymph node dissection as primary surgery. In addition, three biopsies from cervical intraepithelial neoplasia (CIN) patients (CIN III) and three cervical biopsies without epithelial abnormalities were collected. As positive controls, five hyperplastic tonsils were tested.

### Monoclonal and polyclonal antibodies

The anti-TCR ζ monoclonal antibody TIA-2 (Coulter Clone, Almere, NL), recognizing a cytoplasmic epitope of the TCR-associated ζ chain was used to identify ζ chain-positive cells (dilution 1:25). To stain the CD3 complex, the mouse monoclonal antibody Leu4 (Becton Dickinson, San Jose, CA, USA, dilution 1:10), or rabbit polyclonal antibody A542 (DAKO, Glostrup, Denmark, dilution 1:2000), both recognizing the CD3-ε chain, were used.

### Immunohistochemistry

Four 4-μm thick tissue sections were consecutively cut from each specimen. Paraffin sections were mounted on 3% aminopropyltriethoxysilane-coated (Sigma, Deisenhofen, Germany) slides, deparaffinized and rehydrated. After endogenous peroxidase quenching (0.3% hydrogen peroxide methanol for 30 min), antigens were ‘retrieved’ either by boiling sections in sodium citrate buffered solution (0.1 mM, pH 6.0) in a 750 kW microwave oven at maximum power for 10 min (TIA-2) or by preincubating them with 0.1% pronase E (Sigma, Deisenhofen, Germany) for 30 min at 37°C (CD3-ε). No antigen retrieval was used for TIA-2 or CD3-ε staining on frozen sections. Primary antibodies and isotype-matched negative control antibodies at the appropriate dilutions were incubated at room temperature (RT) for 1 h, except for the rabbit polyclonal antibody A542 (O/N, 37°C). A biotinylated secondary antibody to mouse or rabbit immunoglobulins (1:25, 30 min, RT, Dakopatts, Glostrup, Denmark) was followed by a mouse monoclonal antibody directed against biotin (1:200, 30 min, RT, Southern Biotechnology Associates, IN, USA). Complexed streptavidin-biotinylated horseradish peroxidase or alkaline phosphatase was added (1:200 for 1 h, Dakopatts, Glostrup, Denmark) and staining was visualized with diaminobenzidine or new fuchsine on paraffin and frozen sections, respectively. In each experiment, hyperplastic tonsils were included as positive controls.

### Scoring of CD3-ε+ and CD3-ζ+ cells

Slides were examined and quantitatively scored by two independent observers: a total of 100 tumour-infiltrating CD3-ε+ cells were counted in randomly selected high power fields (400 ×) of tumour areas and CD3-ζ+ cells were similarly scored in the same areas on consecutive slides. In lymph nodes, all CD3-ε+ cells were counted present in five HPF of interfollicular spaces or subcapsular sinuses. In the case of tumour-positive lymph nodes, HPF were selected in the proximity of metastasizing tumour nests. For each specimen, the CD3-ζ/ε ratio was calculated and samples were scored as follows: –, $\zeta/\varepsilon = 0$; +, $0 < \zeta/\varepsilon < 0.33$; ++, $0.33 < \zeta/\varepsilon < 0.66$; +++, $0.66 < \zeta/\varepsilon < 1.0$. ζ Chain expression was considered to be down-regulated when the number of CD3-ζ+ cells was less than 33% of the number of CD3-ε+ cells (CD3-ζ/ε <0.33, – or +). When there was disagreement between the two observers, slides were reviewed together until agreement was reached.

### Statistical analysis

Frequencies of CD3-ζ down-regulation were compared between test groups using the Fisher’s exact test. Differences were regarded as significant when $P < 0.05$.

### RESULTS

### CD3-ζ expression in primary cervical carcinomas

CD3+ cells, as determined by staining of the ε chain of the CD3 complex, infiltrating the tumour fields were detectable in all 24 primary tumours studied. The ζ chain expression in the infiltrate was considerably reduced compared to CD3-ε expression (defined by a CD3-ζ/ε ratio of less than 0.33) in 17/24 (71%) of the primary cervical carcinomas. Results obtained for all the studied tumours are summarized in Table 1. Although we only quantified the number of CD3-ζ+ cells within tumour fields, we also observed a reduced ζ chain expression (as compared to CD3-ε) in the stroma surrounding the tumour areas (data not shown). However, this reduction generally appeared to be less severe than the reduction observed in infiltrating cells within the tumour mass. Figures 1C and D show the respective CD3-ε and CD3-ζ expression in

© Cancer Research Campaign 1999

**Table 1** CD3-ζ expression in primary cervical tumours and draining pelvic lymph nodes[a]

| Case no. | Paraffin embedded primary tumour | Frozen primary tumour | Tumour-negative lymph node[b] | Tumour-positive lymph node[b] |
|---|---|---|---|---|
| 1 | + | + | +++ | ND |
| 2 | + | ND | ++ | ND |
| 3 | + | + | ++ | ND |
| 4 | + | + | +++ | +++ |
| 5 | ++ | ++ | ++ | ND |
| 6 | + | ND | +++ | ++ |
| 7 | + | ND | + | +++ |
| 8 | + | ND | +++ | +++ |
| 9 | ND | ND | +++ | +++ |
| 10 | + | ND | +++ | +++ |
| 11 | ++ | ND | +++ | +++ |
| 12 | + | + | ND | ND |
| 13 | + | ND | +++ | ND |
| 14 | + | ND | +++ | ND |
| 15 | + | ND | +++ | ND |
| 16 | ++ | ND | +++ | ND |
| 17 | ++ | ND | ND | ND |
| 18 | ND | + | ND | ND |
| 19 | ND | + | ND | ND |
| 20 | ND | ++ | ND | ND |
| 21 | ND | + | ND | ND |
| 22 | ND | ++ | ND | ND |
| 23 | ND | + | ND | ND |
| 24 | ND | ++ | ND | ND |
| 25 | ND | + | ND | ND |

[a]CD3-ζ expression is classified according to the ratio of the number of CD3-ζ + cells and the number of CD3-ε + cells in the same areas in consecutive tissue sections; –, CD3-ζ/ε = 0; +, 0 < CD3-ζ/ε < 0.33; ++, 0.33 < CD3-ζ/ε < 0.66; +++, 0.66 < CD3-ζ/ε < 1.0, ND = not done. [b]Lymph node biopsies were paraffin-embedded.

lymphocytes infiltrating a primary cervical tumor. A marked reduction of ζ chain expression is apparent. The numeric differences between CD3-ε+ and CD3-ζ+ cells was not due to immunohistochemical artifacts, since in slides with severely reduced ζ chain expression it was always possible to find at least a few CD3-ζ+ cells serving as internal positive controls. Although the necessary microwave retrieval step on paraffin sections resulted in considerably weaker staining for CD3-ζ than for CD3-ε (Figure 1), this did not lead to a bias in the quantitation, as evidenced by results from frozen tissue sections: CD3-ζ staining on frozen sections did not require antigen retrieval and was comparable in intensity to the CD3-ε staining, while similarly reduced CD3-ζ/ε ratios were found for paraffin and frozen sections from the same tumours ($n = 5$, Table 1). CD3-ε and CD3-ζ positive lymphocytes (Figures 1A and B, respectively) are present in equal numbers in a hyperplastic tonsil biopsy. A mean CD3-ζ/ε ratio of 0.9 was found for the total of five hyperplastic tonsils that were tested with only minor variations between the different tonsil biopsies. These tonsils served as positive controls in all performed tests. We also determined CD3-ζ expression in three premalignant cervical intraepithelial neoplastic (CIN) lesions (CIN III), which are regarded as the direct precursor lesions of cervical carcinoma, and in three cervical biopsies without apparent epithelial abnormalities. We observed considerably reduced CD3-ζ expression in infiltrating lymphocytes in two out of three CIN III lesions (CD3-ζ/ε <0.33) with the remaining CIN III lesion showing a less pronounced reduction of CD3-ζ expression (0.33 < CD3-ζ/ε <0.67). The biopsies with normal cervical epithelium were harder to analyse due to the absence of lymphocytes infiltrating the squamous epithelial layers. However, no difference in the amount of CD3-ε+ and CD3-ζ+ cells was detected in T-cells present in the stroma directly underneath the epithelial basal layer.

### CD3-ζ expression in tumour-positive and tumour-negative pelvic lymph nodes

A total of 15 tumour-negative and seven tumour-positive pelvic lymph nodes were examined for CD3-ε and ζ expression. In 11 out of 15 tumour-negative lymph nodes CD3-ε and CD3-ζ were equally expressed, while only in one biopsy a considerably reduced CD3-ζ expression was observed (CD3-ζ/ε < 0.33). In tumour-positive lymph nodes, tumour nests were usually surrounded by a thick band of CD3-ε+ cells, with some T-cells intermingled with the tumour cells. In all seven tumour-positive lymph nodes studied, CD3-ζ expression was strong and comparable to CD3-ε expression (+++ in 6/7 and ++ in 1/7 cases, see Table 1). Also on lymphocytes in between metastasizing tumour cells a good CD3-ζ expression was observed. CD3-ε and CD3-ζ expression in a tumour-positive lymph node are shown in Figure 1E and F, respectively.

CD3-ζ expression in tumour-positive and tumour-negative lymph nodes derived from the same patient was mostly comparable, except for case no. 7 where a reduced CD3-ζ expression (comparable to the expression in the primary tumour) was observed in the tumour-negative lymph node, while CD3-ε and CD3-ζ were equally expressed in the tumour-positive lymph node (Table 1).

From 14 patients both the primary tumour and tumour-negative lymph nodes were available. In addition, tumour-positive lymph nodes were also available from six of these patients (see Table 1). The frequency of CD3-ζ loss (CD3-ζ/ε < 0.33) in the primary

© Cancer Research Campaign 1999 

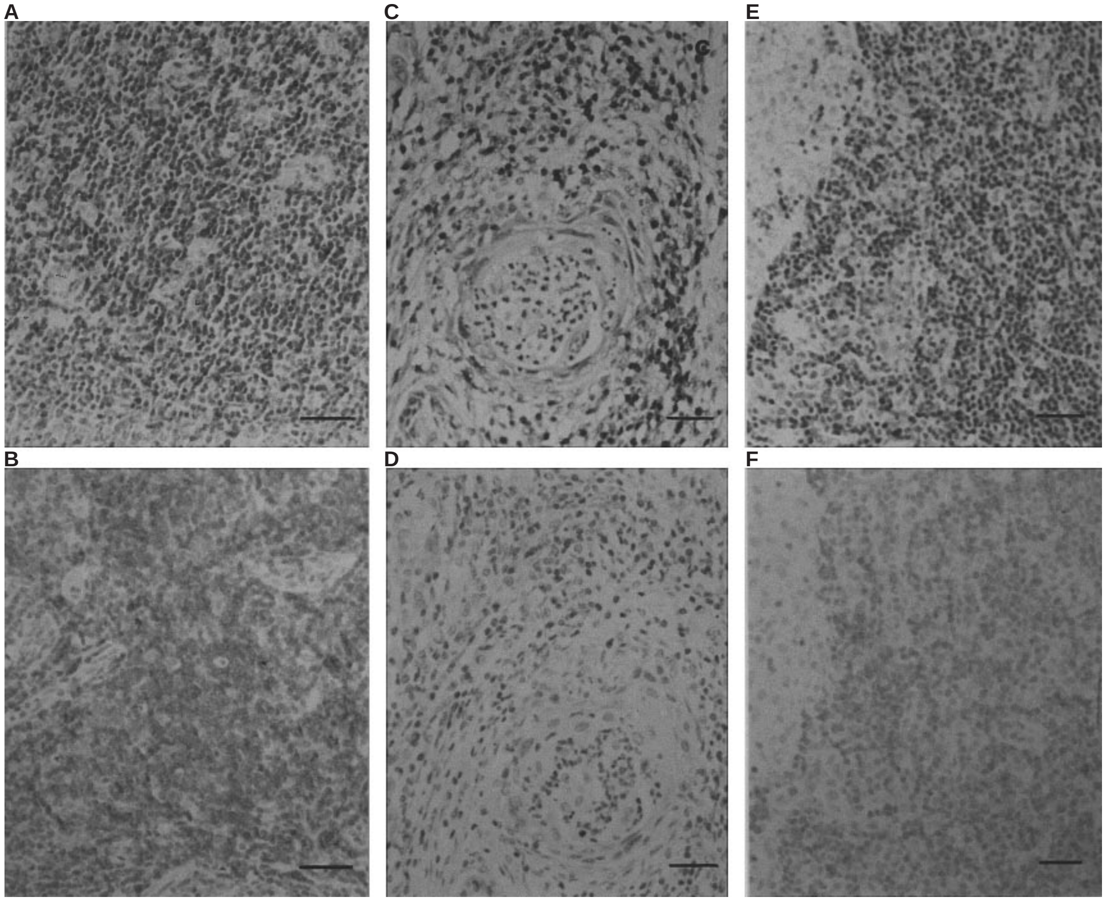

**Figure 1** CD3-ε and CD3-ζ expression in a hyperplastic tonsil (**A** and **B**, respectively), a primary cervical tumour (**C** and **D**), and in a corresponding metastasis-positive lymph node (**E** and **F**). CD3-ε staining (**A**, **C** and **E**) was performed with the polyclonal antibody CD3 A542 and CD3-ζ (**B**, **D**, and **F**) with the monoclonal antibody TIA-2. Size bars represent 100 μm tumours was significantly higher (11/14, 79%) than in the corresponding tumour-negative lymph nodes (1/14, 7%, $P < 0.0005$). Similarly, the frequency of CD3-ζ loss was higher in the primary tumours (5/6, 83%) as compared to the corresponding metastasis-positive draining lymph nodes (0/6, 0%, $P < 0.02$). Also, the frequency of CD3-ζ down-regulation in the total of the tested primary carcinomas (17/24, 71%) was significantly higher than the frequency of down-regulation in all the lymph nodes (both tumour-positive and tumour-negative) studied (1/22, 4.5%, $P < 0.00001$, Figure 2).

### DISCUSSION

In this study we report a putative local impairment of cellular immune functions in primary cervical carcinoma due to the loss of the signal transducing ζ chain from the TCR/CD3 complex of tumour-infiltrating lymphocytes. In the majority of our carcinoma samples, a down-regulation of CD3-ζ expression was observed as compared to the expression of CD3-ε in the same tumour fields on consecutive slides. In contrast, CD3-ζ expression was retained in T-cells surrounding metastatic deposits in draining pelvic lymph nodes and also in tumour-negative lymph nodes.

The difference in expression levels between tumours and draining lymph nodes suggests that the observed loss of CD3-ζ expression is caused by factors released from the primary tumour

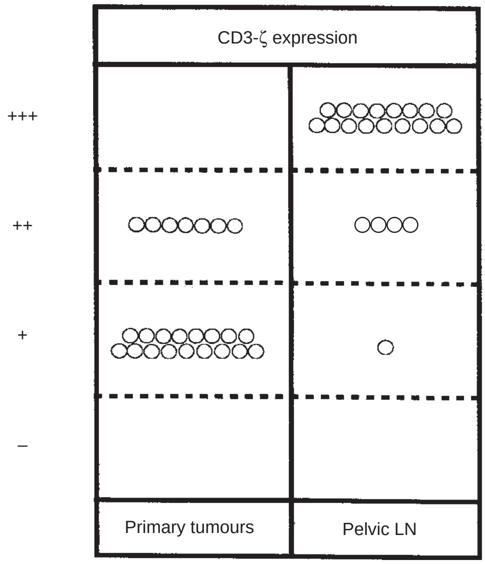

**Figure 2** CD3-ζ expression levels in primary cervical carcinomas (prim. tumours, $n = 24$) and in both tumour-negative ($n = 15$) and in tumour-positive ($n = 7$) draining pelvic lymph nodes (pelvic LN, total: $n = 22$). CD3-ζ expression is classified according to the ratio of the number of ζ+ cells and the number of CD3-∊ + cells in the same areas in consecutive tissue sections; -: CD3-ζ/∊ = 0, +: 0 < CD3-ζ/∊ < 0.33, + +: 0.33 < CD3-ζ/∊ < 0.66, + + +: 0.66 < CD3-ζ/∊ < 1.0. CD3-ζ expression was considered to be lost in case of – or + classification (CD3-ζ/∊ < 0.33)

© Cancer Research Campaign 1999

and may be a localized effect. This is in keeping with previous observations in colon carcinoma where CD3-ζ loss on mucosa-infiltrating T-cells was found to be inversely correlated to the distance from the primary tumour site (Matsuda et al, 1995). However, employing FACS analysis, a down-regulation of CD3-ζ has also been reported on peripheral lymphocytes from cervical carcinoma patients (Kono et al, 1996*a*), indicating that this is not merely a local effect. The latter study did not include an analysis of tumour-infiltrating lymphocytes, but our study indicates that these cells would have been likely to have had a more pronounced loss of CD3-ζ expression. NK-cells can also express ζ chains which associate with the low-affinity (type III) Fcγ receptor (CD16) (Kiessling et al, 1996). Indeed, besides loss of CD3-ζ from T-cells, Kono et al (1996*a*) also showed down-regulation of ζ chains on NK-cells in the peripheral blood of cervical carcinoma patients. By staining carcinoma sections for the NK-cell marker CD56, we found relatively few NK-cells infiltrating the tumour areas (data not shown). We therefore conclude that the observed change in ζ chain expression in cervical tumours is mainly due to the modulation of CD3-ζ expression on tumour-infiltrating T-cells.

Our observation of putatively impaired T-cell functions in cervical neoplasia by ζ chain down-regulation may seem to conflict with our recently published findings of an increase in granzyme B expression by CD8+ T-cells in cervical carcinoma, which is a clear sign of cytotoxic T-cell (CTL) activation (Bontkes et al, 1997). However, in early stage colon carcinomas (Duke's A) we previously reported a similar increase in granzyme positivity with a simultaneously decreased ζ chain expression (Mulder et al, 1997). It seems likely that, in tumours, the degree of T-cell activation on the one hand and T-cell suppression on the other, will depend on the local balance between the availability of tumour antigens accompanied by appropriate pro-inflammatory signals and tumour-derived immunosuppressive factors (Matzinger, 1994; Chouaib et al, 1997). To further study the direct relationship between ζ chain expression and the ability of T-cells to express granzyme B, a double-staining method for these markers will have to be developed.

The mechanisms that are responsible for ζ chain loss in tumour-infiltrating T-cells remain largely unclear. Experiments in tumour-bearing mice have shown that the levels of ζ chain mRNA were not affected but that proteolysis of the ζ chain was increased. Lysosomal degradation was implicated as the cause of this increased rate of ζ chain turnover (Correa et al, 1997). Immunosuppressive cytokines such as IL-10 and TGF-β, possibly tumour-derived, might seem obvious causative agents, but were shown to have no effect on CD3-ζ expression in vitro (Kiessling et al, 1996). Recently, hydrogen peroxide derived from tumour-associated macrophages was found to down-modulate CD3-ζ expression on peripheral lymphocytes (Kono et al, 1996*b*). Interestingly, macrophages isolated from lymph nodes with melanoma metastases were shown to inhibit CD3-ζ expression through contact-dependent mechanisms (Kiessling et al, 1996). Whether macrophage-derived factors are the main causative agents responsible for the in vivo loss of CD3-ζ expression remains to be established.

There are several possible explanations for the difference in CD3-ζ expression we observed between primary cervical carcinomas and metastasis-positive lymph nodes: a differential expression of tumour- or macrophage-derived factors with ζ chain modulating capacities in primary tumours and metastases, higher levels of IL-2 in lymph nodes which may restore CD3-ζ expression (Kiessling et al, 1996), or a higher turnover rate of T-cells in lymph nodes as compared to T-cells infiltrating the primary tumour. A murine study demonstrated that T-cells must be exposed for at least 26 days to the putative tumour-released factor, in order for the CD3-ζ chain to be down-regulated (Mizoguchi et al, 1992). Thus, a rapid turnover of T-cells in the lymph nodes might protect them from ζ chain loss.

Kono et al (1996*a*) reported that the loss of CD3-ζ expression on peripheral lymphocytes was already apparent in patients with premalignant cervical lesions. This is in keeping with our finding of a reduced CD3-ζ expression on epithelium-infiltrating lymphocytes in two out of three tested CIN III lesions. It remains to be established whether decreased CD3-ζ expression is directly correlated to the progression of cervical neoplastic lesions or rather is a consequence of HPV infections per se. It is conceivable that CD3-ζ down-modulation is a reaction to chronic antigenic stimulation resulting from persistent HPV infections. In that case, CD3-ζ loss might affect HPV-specific T-cell tolerance as has been hypothesized for other pathogen- or tumour-specific immune responses (Levey and Srivastava, 1996). This notion is in keeping with the observed loss of CD3-ζ in such a chronic inflammatory condition as rheumatoid arthritis (Kiessling et al, 1996; Maurice et al, 1998). Whether only a generalized or also a specific effect, in either case the loss of CD3-ζ from the signal transduction machinery of T-cells is likely to hamper effective cellular immune responses to HPV-transformed keratinocytes in (pre)malignant cervical lesions.

In conclusion, reduced CD3-ζ expression on T-cells infiltrating primary cervical carcinomas may result in an impaired cell-mediated anti-tumour response. In order for vaccination strategies to be successful, it may be essential to first identify and counteract mechanisms leading to this loss of CD3-ζ.

### ACKNOWLEDGEMENTS

F Peccatori has been a recipient of a fellowship from the European Cancer Centre, Amsterdam. This work was supported by the Prevention Fund (28-1502.2) and the University Stimulation Fund (USF) of the Free University.

### REFERENCES

Bontkes HJ, de Gruijl TD, Walboomers JMM, van den Muysenberg AJC, Gunther AW, Scheper RJ, Meijer CJLM and Kummer JA (1997) Assessment of cytotoxic T-lymphocyte phenotype using the specific markers granzyme B and TIA-1 in cervical neoplastic lesions. *Br J Cancer* **76**: 1353–1360

Chouaib S, Asselin-Paturel C, Mami-Chouaib F, Caignard A and Blay JY (1997) The host-tumor immune conflict: from immunosuppression to resistance and destruction. *Immunol Today* **18**: 493–497

Correa MR, Ochoa AC, Ghosh P, Mizoguchi H, Harvey L and Longo DL (1997) Sequential development of structural and functional alterations in T cells from tumor-bearing mice. *J Immunol* **158**: 5292–5296

Cromme FV, Meijer CJLM, Snijders PJF, Uyterlinde A, Kenemans P, Helmerhorst T, Stern PL, Vandenbrule AJC and Walboomers JMM (1993) Analysis of MHC class-I and class-II expression in relation to presence of HPV genotypes in premalignant and malignant cervical lesions. *Br J Cancer* **67**: 1372–1380

Cromme FV, Walboomers JMM, Stukart MJ, de Gruijl TD, Kummer JA, Leonhart AM, Helmerhorst TJM and Meijer CJLM (1995) Lack of granzyme expression in T lymphocytes indicates poor cytotoxic T lymphocyte activation in human papillomavirus-associated cervical carcinomas. *Int J Gynecol Cancer* **5**: 366–373

de Gruijl TD, Bontkes HJ, Stukart MJ, Walboomers JMM, Remmink AJ, Verheijen RHM, Helmerhorst TJM, Meijer CJLM and Scheper RJ (1996*a*) T cell proliferative responses against human papillomavirus type 16 E7 oncoprotein are most prominent in cervical intraepithelial neoplasia patients with a persistent viral infection. *J Gen Virol* **77**: 2183–2191

© Cancer Research Campaign 1999 

de Gruijl TD, Bontkes HJ, Walboomers JMM, Stukart MJ, Robbesom AAJP, von Blomberg-van der Flier BME, Herbrink P, Remmink AJ, Verheijen RHM, Helmerhorst TJM, Meijer CJLM and Scheper RJ (1996*b*) Analysis of IgG reactivity against human papillomavirus type-16 E7 in patients with cervical intraepithelial neoplasia indicates an association with clearance of viral infection: results of a prospective study. *Int J Cancer* **68**: 731–738

Finke JH, Zea AH Longo DL, Mizoguchi H, Tubbs RR, Wiltrout RH, O'Shea JJ, Kudou S, Klein E, Bukowski RM and Ochoa A (1993) Loss of T-cell receptor zeta chain and P56lck in T-cells infiltrating human renal cell carcinoma. *Cancer Res* **53**: 5613–5616

Frazer IH (1996) Immunology of papillomavirus infection. *Curr Op Immunol* **8**: 484–491

IARC (1995) *Monographs on the Evaluation of the Carcinogenic Risks to Humans, vol. 64, The Human Papillomavirus*. International Agency for Research on Cancer: Lyon

Kiessling R, Kono K, Petersson M and Wasserman K (1996) Immunosuppression in human tumor-host interaction: role of cytokines and alterations in signal-transducing molecules. *Springer Sem Immunopatol* **18**: 227–242

Kono K, Ressing ME, Brandt RMP, Melief CJM, Potkul RK, Andersson B, Petersson M, Kast WM and Kiessling R (1996*a*) Decreased expression of signal-transducing zeta chain in peripheral T cells and natural killer cells in patients with cervical cancer. *Clin Cancer Res* **2**: 1825–1828

Kono K, Salazar-Onfray F, Petersson M, Hansson J, Masucci G, Wasserman K, Nakazawa T, Andersson P and Kiessling R (1996*b*) Hydrogen peroxide secreted by macrophages/monocytes inhibits tumor specific T-cell and NK mediated cytotoxicity and down-regulates expression expression of signal-transducing molecules. *Eur J Immunol* **26**: 1308–1313

Lai P, Rabinowich H, Crowley-Nowick PA, Bell MC, Mantovani G and Whiteside TL (1996) Alterations in expression and function of signal-transducing proteins in tumour-associated T and natural killer cells in patients with ovarian carcinoma. *Clin Cancer Res* **2**: 161–173

Levey DL and Srivastava PK (1996) Alterations in T cells of cancer-bearers: whence specificity? *Immunol Today* **17**: 365–368

Matsuda M, Petersson M, Lenkei R, Taupin J-L, Magnusson I, Mellstedt H, Andersson P and Kiessling R (1995) Alterations in the signal-transducing molecules of T cells and NK cells in colorectal tumor-infiltrating, gut mucosal and peripheral lymphocytes: correlation with the stage of the disease. *Int J Cancer* **61**: 765–772

Matzinger P (1994) Tolerance, danger and the extended family. *Annu Rev Immunol* **12**: 991–1045

Maurice MM, Lankester AC, Bezemer AC, Geertsma MF, Tak PP, Breedveld FC, van Lier RAW and Verweij CL (1998) Defective TCR-mediated signaling in synovial T cells in rheumatoid arthritis. *J Immunol* **159**: 2973–2978

Mizoguchi H, O'Shea JJ, Longo DL, Loeffler CM, McVicar DW and Ochoa A (1992) Alterations in signal transduction molecules in T-lymphocytes from tumor bearing mice. *Science* **258**: 1795–1798

Mulder WMC, Bloemena E, Stukart MJ, Kummer JA, Wagstaff J and Scheper RJ (1997) T cell receptor-zeta and granzyme B expression in mononuclear cell infiltrates in normal colon mucosa and colon carcinoma. *Gut* **40**: 113–119

Nakagomi H, Petersson M, Magnusson I, Juhlin C, Matsuda M, Mellstedt H, Taupin J-L, Vivier E, Anderson P and Kiessling R (1993) Decreased expression of the signal-transducing zeta chains in tumor-infiltrating T-cells and NK cells of patients with colorectal carcinoma. *Cancer Res* **53**: 5610–5612

Robey E and Allison JP (1995) T-cell activation: integration of signals from the antigen receptor and costimulatory molecules. *Immunol Today* **16**: 306–310

Rosenberg AS (1996) Development of cancer immunotherapies based on identification of the genes encoding cancer regression antigens. *J Natl Cancer Inst* **88**: 1635–1644

Tartour E, Latour S, Mathiot C, Thiounn N, Mosseri V, Joyeux I, D'Enghien CD, Lee R, Derbre B and Fridman WH (1995) Variable expression of CD3-zeta chain in tumor-infiltrating lymphocytes (TIL) derived from renal-cell carcinoma: relationship with TIL phenotype and function. *Int J Cancer* **63**: 205–212

Whiteside TL, Jost LM and Herbermann RB (1992) Tumor-infiltrating lymphocytes. Potential and limitations to their use for cancer therapy. *Crit Rev Oncol Hematol* **12**: 25–47

Zea AH, Curti BD, Longo CD, Alvord WG, Srobl SL, Mizoguchi H, Creekmore SP, O'Shea JJ, Powers GC, Urba WJ and Ochoa AC (1995) Alteration in T cell receptor and signal transduction molecules in melanoma patients. *Clin Cancer Res* **1**: 1327–1335

*© Cancer Research Campaign 1999*
